# An explorative study identifies miRNA signatures for the diagnosis of non-celiac wheat sensitivity

**DOI:** 10.1371/journal.pone.0226478

**Published:** 2019-12-13

**Authors:** Emanuela Clemente, Konstantinos Efthymakis, Erminia Carletti, Vanessa Capone, Samantha Sperduti, Giuseppina Bologna, Marco Marchisio, Marta Di Nicola, Matteo Neri, Michele Sallese

**Affiliations:** 1 Department of Medical, Oral and Biotechnological Sciences, “G. d'Annunzio” University of Chieti–Pescara, Chieti, Italy; 2 Centre for Advanced Studies and Technology (CAST), “G. d'Annunzio” University of Chieti-Pescara, Chieti, Italy; 3 Department of Medicine and Ageing Sciences, “G. d'Annunzio” University of Chieti–Pescara, Chieti, Italy; University of Oslo, NORWAY

## Abstract

Non-celiac wheat sensitivity (NCWS), also referred to as non-celiac gluten sensitivity, is a recently described disorder triggered by wheat/gluten ingestion. NCWS elicits a wide range of symptoms including diarrhoea, intestinal discomfort, and fatigue in analogy with other wheat/gluten-related disorders and celiac disease in particular. From the pathological standpoint, NCWS patients only have a slight increase of intraepithelial lymphocytes, while antibodies to tissue transglutaminase (tTG) and villous atrophy, otherwise diagnostic features of celiac disease, are absent. To date, the diagnosis of NCWS relies on symptoms and exclusion of confounding diseases, since biomarkers are not yet available. Here, the expression levels of selected miRNAs were examined in duodenal biopsies and peripheral blood leukocytes collected from newly diagnosed patients with NCWS and, as controls, from patients with celiac disease and gluten-independent gastrointestinal problems. We identified a few miRNAs whose expression is higher in the intestinal mucosa of patients affected by NCWS in comparison to control patients affect by gluten-independent dyspeptic symptoms (*Helicobacter pylori*-negative) and celiac disease. The present study provided the first evidence that NCWS patients have a characteristic miRNA expression patterns, such peculiarity could be exploited as a biomarker to the diagnosis of this disease.

## Introduction

Wheat is the basis of human nutrition, yet the consumption of specific components of wheat, including gluten, amylase trypsin inhibitor, (ATI) and fermentable oligo-, di-, monosaccharide, (FODMAPs) can cause abdominal pain, bloating, vomiting, diarrhoea, constipation, headache, tiredness, urticarial, dermatitis, numbness, neuropsychiatric disorders malabsorption of nutrients and ataxia [1–4]. Most of these symptoms are common to different wheat/gluten-related disorders including wheat allergy, celiac disease (CD) and the recently described non-celiac wheat sensitivity (NCWS) [3, 5–8].

CD, the best know gluten-related disorder, is an inflammatory disease induced by gluten ingestion in genetically susceptible individuals. Incompletely digested gluten peptides can reach the lamina propria and trigger an adaptive cellular and humoral immune response in people expressing the human leukocyte antigen (HLA) class II DQ2 or DQ8 [6, 9–12]. Lymphocytes infiltrate the intestinal epithelium, crypts become hyperplastic and villi undergo atrophy, overall profoundly affecting the architecture and physiology of the intestinal mucosa. CD is suspected based on serological markers, mainly IgA antibodies anti-tTG and anti-endomysial (EmA) [4]. However, alteration of the mucosal architecture, according to Marsh classification, is necessary to confirm the diagnosis [13–16].

When wheat ingestion causes distress in patients with preserved intestinal architecture and in the absence of wheat allergy, NCWS can be hypothesized [3, 6]. The pathogenic mechanism of NCWS is poorly defined and the diagnosis is based on symptoms and exclusion criteria as biomarkers are not available [6, 17, 18]. Adaptive cytokines that typically increase in CD (e.g. IL6, IL21, interferon gamma, IL17A) does not seem to be involved in NCWS [19]. Some studies suggested that gluten-dependent activation of innate immunity could be involved in NCWS [18, 19]. The over-expression of Toll like receptor 1 (TLR1), TLR2 and the down regulation of the T regulatory cell marker FoxP3 has been reported in the small intestine mucosa of these patients[19]. Furthermore, serum from patients with NCWS showed increased levels of lipopolysaccharide (LPS)-binding protein, soluble CD14, and antibodies against LPS and flagellin [18]. The prevalence of NCWS is still unclear, although approximately 6% of the 5896 patients seen at Center for Celiac Research, University of Maryland from 2004 to 2010, were diagnosed with NCWS [6]. While, of the 7762 non-institutionalised people surveyed by the National Health and Nutrition Examination Survey (NHANES) only 0.6% spontaneously followed a gluten-free diet [20]. This behaviour was considered a surrogate of NCWS prevalence in the general population [20]. However, the most recent estimate suggests a prevalence of about 1% among the general population [21].

The correct identification of patients affected by NCWS is important to provide appropriate treatments/handling to these patients. Gene expression profiles have shown to have the necessary qualification to carry on this endeavour [22, 23].

Mammalian cells produce a few thousands of miRNAs, short (20–25 nucleotides) RNA sequences that regulate the expression of many genes and virtually control all cellular functions [23–26]. MiRNAs are initially transcribed by RNA polymerase II in the form of primary RNA (pri-miRNA), processed by the ribonuclease III Drosha in small fragments of 65–70 nucleotides with a hairpin structure. Then, they are transported into the cytoplasm and further remodelled into mature miRNAs by a ribonuclease III called Dicer [27]. Mature double-stranded miRNA binds to the RNA-Induced Silencing Complex (RISC), which select one of the two strands. The selected strand often referred as “guide RNA” is the functional miRNA, the other filament, the "passenger", is discarded. The miRNA-RISC complex patrols the cytoplasm in search of target mRNAs. These mRNAs undergo cutting and degradation by RISC and other ribonucleases with the result to reduce the availability of mRNA for translation. To date, miRNA have been involved in autoimmune diseases, innate immune response, and differentiation of intestinal epithelium [24]. Furthermore, different studies reported differentially expressed miRNA that are potentially helpful to the diagnosis of CD [25, 28–30]

In the present study, we evaluated the expression of a collection of miRNAs in the intestinal mucosa of NCWS patients and controls affected by gluten-independent dyspeptic symptoms (*Helicobacter pylori*-negative) and CD. We identified differentially expressed miRNA whose expression is potentially helpful to the diagnosis of patients affected by NCWS.

## Materials and methods

### Design of the study and patients’ recruitment

Patients referred to the Regional Centre for Adult Celiac Disease and the Gastroenterology and Endoscopy Unit at the “SS. Annunziata” University Hospital of Chieti claiming gluten-related symptoms were examined according to the current clinical practice. Physical examination was performed; medical history for recent or chronic disease, prior surgery and current drug use was collected. As per study protocol, patients expressing the HLA-DQ2/DQ8 haplotypes, showing positive CD serology (IgA anti-tTG and/or anti-EmA), and villous atrophy at histology (mostly Marsh grade ≥3) were enrolled as CD (Table 1).

**Table 1:** Patients’ characteristics.

	CTRL	NCWS	CD	p-value
**Pilot cohort (biopsies)**				
Number of subject	17	13		
Age (yr), *mean±SD*	55.3±20.0	41.3±12.4		*0*.*035*^*a*^
Gender (M/F)	10/14	3/24		*0*.*029*^*b*^
Marsh-classification, *n(%)*				*-*
0		6 (46.1)		
1		7 (53.8)		
tTG-IgA, (U/ml) *mean±SD*	2.1±0.9	2.0±1.1		*0*.*786* ^*a*^
IEL/100 epithelial cells, *mean±SD*	-	24.3±6.5		*-*
HLA-DQ				
Negative		8 (61.5)		
DQ2		4 (30.8)		
DQ8		1 (7.7)		
**Validation cohort (biopsies)**				
Number of subject	25	27	24	
Age (yr), *mean±SD*	54.7±19.7	41.1±12.3	39.5±16.3	*0*.*002*^*c*^
Gender (M/F)	10/15	3/24	5/19	*0*.*052*^*b*^
Marsh-classification, *n(%)*				*<0*.*001*^*b*^
0		15 (55.6)	-	
1		12 (44.4)	4 (16.7)	
3a		-	8 (33.3)	
3b		-	5 (20.8)	
3c		-	7 (29.2)	
tTG-IgA, (U/ml) *mean±SD*	2.30±0.9	2.5±2.6	124.8±109.1	*<0*.*001* ^*c*^
IEL/100 epithelial cells, *mean±SD*	-	22.8±9.7	49.4±18.2	*<0*.*001* ^*a*^
HLA-DQ				
Negative		17 (63.0)	-	*<0*.*001* ^*b*^
DQ2		8 (29.6)	22 (91.7)	
DQ8		2 (7.4)	2 (8.3)	
**Validation cohort (PBL)**				
Number of subject	21	19		
Age (yr), *mean±SD*	51.2±20.1	41.8±13.3		*0*.*093*^*a*^
Gender (M/F)	6/15	3/16		*0*.*557*^*b*^
Marsh-classification, *n(%)*				
0		9 (47.4)		
1		10 (52.6)		
3a		-		
3b		-		
3c		-		
tTG-IgA, (U/ml) *mean±SD*	1.9±0.9	3.5±3.1		*0*.*029* ^*a*^
IEL/100 epithelial cells, *mean±SD*	-	23.4±9.9		*-*
HLA-DQ				
Negative		13 (68.4)		
DQ2		6 (31.6)		
DQ8		-		

^a^ Student t-test for unpaired data

^b^ chi-squared test or Fisher exact test when appropriate

^c^one-way ANOVA test. IEL = intraepithelial lymphocytes.

Patients presenting with gastrointestinal symptoms, a negative CD serology (IgA anti-tTG and/or anti-EmA), showing normal total IgA titres and preserved mucosal architecture (Marsh grade ≤1), were enrolled as potential NCWS, independently from HLA haplotype. Serologically negative patients with HLA-DQ2/8 positivity were purposefully excluded from the NCWS group, when a positive family history for celiac disease among 1st degree relatives was reported. Furthermore, subjects were required to have negative immuno-allergy tests to wheat, a negative glucose hydrogen breath test to exclude small intestinal bacterial overgrowth (SIBO), and to show a resolution of symptoms after a non-blinded 6-week gluten exclusion period, with subsequent positive open gluten challenge. The open gluten-free diet was prescribed by an experienced nutritionist for a 6 week-period, after which persistently symptom-free subjects were reintroduced to dietary wheat protein (equivalent to 10 gr of gluten). Only then, symptom recurrence prompted a diagnosis of non-celiac wheat/gluten sensitivity [6, 19, 31]. Symptom severity was assessed using a modified diagnostic questionnaire (Gastrointestinal Symptom Rating Scale) at baseline and after gluten exclusion. The right definition and recruitment of the patients is a main issue in this kind of studies as NCWS is ill-defined condition that could be triggered by gluten and other wheat components like FODMAPs or non-gluten proteins like ATI [1, 2]. Changing the diet to remove the gluten also modifies the amounts of FODMAPs and non-gluten proteins. Therefore, the enrolled patients should be referred to as NCWS because other components of wheat besides gluten may have contributed to fluctuations of symptoms.

Finally, *Helicobacter pylori*-negative patients with gluten-independent dyspeptic symptoms who required endoscopic examination were also recruited as controls (Table 1). Dyspepsia was defined according to Rome III criteria, in addition to a negative EGD, H. pylori and SIBO status. We specifically included patients with epigastric burning, as their primary manifestation. Patients reported no association of symptoms to specific dietary components at initial inclusion. Those who accepted an elimination diet as an initial treatment strategy were prescribed a gluten-free dietary scheme by an experienced nutritionist for 4 weeks. Controls were chosen among the non-responders to the gluten free diet (GFD).

All these patients underwent upper endoscopy within one month from laboratory assessment. At least five duodenal biopsies were obtained for histological examination, including one in the duodenal bulb. Narrow Band Imaging and white light magnification were used to aid biopsy sampling. Biopsy samples were placed on cellulose paper to maintain orientation and prevent artefacts. An additional biopsy destined for inclusion in this study was taken from the immediate vicinity of those intended for histological examination. The endoscopist, based on endoscopic presentation, determined the actual site. Biopsy and peripheral blood samples used in the study were obtained prior to any gluten exclusion diet.

Ethical approval was obtained from the Clinical Research Ethics Committee dell’Università degli Studi “G. D’Annunzio” and ASL N°2 Lanciano-Vasto-Chieti; Report number 13, 07/18/2013. Study participants gave written informed consent prior to inclusion. Furthermore, all experiments and methods were performed in accordance with relevant guidelines and regulations.

### Processing of patient specimens and evaluation of miRNA expression

Freshly isolated duodenal biopsies were submerged in RNAlater^™^ and stored at -80°C until RNA extraction. Peripheral blood was collected into EDTA-containing tubes and peripheral blood leukocytes (PBL) isolated using the polymorphprep buoyant density gradient according to manufacturer’s indications (Axis‐Shield) and as previously described [32]. Polymorphonuclear and mononuclear cells were pooled together, lysed in QIAzol and immediately frozen at -80°C until RNA extraction.

Total RNA was extracted from biopsies and PBL using the miRNeasy Kit from Qiagen according to manufacturer’s indications. This Kit enables purification of RNA from approximately 18 nucleotides upwards.

The analysis of miRNA expression was carried out in subsequent steps.

1) The expression of 136 miRNA was evaluated in the RNA extracted from the duodenal biopsies of a small group (pilot cohort) of NCWS and control patients affected by gluten-independent dyspeptic symptoms. One hundred ng of total RNA was retro-transcribed using the miScript II RT Kit (Qiagen) and amplified (1 ng/well) using the miScript miRNA PCR Arrays named ‘Immunopathology’ and ‘Inflammatory Response & Autoimmunity’ (Qiagen) according to the manufacturer instructions. The miRNA included in the “Immunopathology” array were selected on the bases of literature data showing their differential expression between normal and pathological immune responses. The miRNA included in the “Inflammatory Response & Autoimmunity” array were selected on the bases of their putative ability to regulate genes relevant to inflammation and autoimmune diseases by using in-silico approaches.

The Expression of miRNAs were normalised using the following five small nucleolar RNA SNORD61, SNORD68, SNORD72, SNORD95 and SNORD96a and one small nuclear RNA RNU6-2. These references RNA are rather stable among tissues and cell types and have an amplification efficiency close to 100% (https://www.qiagen.com/us/shop/pcr/primer-sets/miscript-pcr-controls/). They were widely used to normalise miRNA expression by qPCR[33],[34],[35]. We decided to use multiple reference points because a single one could unexpectedly change and alter the data, while the average of multiple references is more robust and resistant to perturbations.

Thermal cycles were as follow: 15 min at 95°C to activate the Taq polymerase, and 40 cycles of 94°C for 15 sec, 55°C for 30 sec and 70°C for 30 sec.

2) The expression levels of the miRNAs identified as differential in the first step were validated in a large number of patients (validation cohort) using a manually set quantitative real-time PCR. Briefly, total RNA from duodenal biopsies was retrotranscribed as described above and amplified (1 ng/well) using commercially available miScript Primer Assay (Qiagen). Extensive experimental validation (https://www.qiagen.com/us/shop/pcr/primer-sets/miscript-primer-assays/) showed that "miScript Primer Assay" are very sensitive (as low as 10 copies of miRNA can be detected), have a broad dynamic range, a high efficiency of amplification and are highly specific even when miRNAs differ for a single base. The “in-house PCR” amplifications were normalised using SNORD95 and RNU6-2 described above as well as the validated SNORD44 [36, 37]. Thermal cycles were as above.

3a) The expression levels of the miRNAs identified in the first step were examined in duodenal biopsies of patients affected by CD following the protocol used in the second step.

3b) The expression levels of the miRNAs identified in the first step were examined in PBL of NCWS and control patients affect by gluten-independent dyspeptic symptoms according to the protocol described in the second step.

### Statistical analysis

The quantitative variables were summarized as mean ± standard deviation (SD) in the tables. The qualitative variables were summarized as frequency and percentage. The results were reported separately for each groups. Shapiro-Wilk’s test was performed to verify normal distribution of data. Statistical significance of differences between groups for qualitative variables were assessed using the Chi-squared test or Fischer’s Exact Test, when appropriate. Student’s t test for unpaired data or one-way Analysis of Variance (ANOVA) was applied for assessing the comparison of the quantitative variables between groups.

Amplifications were normalised for the effective amount of RNA used in the reaction by calculating the delta Ct (ΔCt), that is the Ct of the miRNA under investigation subtracted of the mean Ct of the reference RNA (the small nucleolar RNA described above). A Shapiro-Wilk’s test was performed to evaluate the departures from normality distribution of ΔCt values. Since the distributions of ΔCt values were not significantly different from normal distribution parametric methods were applied. Fold change (FC) differences in miRNA expression between NCWS and control groups were calculated using the ΔΔCt method [38]. To give an indication of the precision of the FC, the 95% confidence interval was determined.

To select differentially expressed miRNAs between NCWS and control groups, linear regression models were performed with ΔCt values as dependent variable and each group as the independent variable, adjusting for age and gender. Previous studies reported that adjusting for these parameters using statistical corrections gives results similar than matching patients for age and gender [39], nevertheless this remain a critical point of this study. Volcano plot visualization based on FC and p-values of the linear regression was performed (FC≥1.5 and p-value ≤0.05). Finally, to take into account for multiple comparisons, a False Discovery Rate (FDR) correction of 5% was applied [40].

Differentially expressed miRNA selected in the pilot phase, were further analysed on the validation cohort that also included the CD patient group. According to our estimate, the minimum sample size required for the validation study based on the results obtained on the pilot cohort was 22. Twenty-two observations for each group is considered necessary to achieve a power of 80% with a Type I error of 0.05, to detect a statistically significant difference of 0.5 with a sigma of 0.4 for each planned comparison with FDR at 5% [41]. After Shapiro-Wilk’s test, the linear regression models (ANOVA), adjusted for age and gender, were performed on ΔCt values to evaluate significance of difference among the three groups (CTRL, NCWS and CD). Dunnett's t post-hoc test were applied to evaluate pairwise significance of difference between groups. FC and relative 95% confidence interval of miRNA expression between groups were calculated as above. Note that, FC and FDR filtering were not applied in the validation phase.

The linear discriminant analysis with cross validation was performed to classified patients into different groups, according to differentially expressed miRNAs. The classification of a patient is based on the combination of *prior probabilities* with *discriminant functions*, which assign a *score* to each group. The case is then assigned to the group with highest score.

The principal component analysis (PCA), which is suitable for the regression of high-dimensional data, was performed on the ΔCt values. miRNAs with component score coefficient matrix ≥0.4 or ≤0.4 were selected. PCA scores were calculated using the covariance matrix, and principal components 1 and 2 (PC1 and PC2) were determined to explain most of the data variation (Scree test). PC1 was plotted against PC2 to identify miRNA driving the two components and to explain the percentage of variance. These associations were tested formally using a logistic regression analysis with the principal components as the exposure variables estimating the odds ratio (OR) and the relative 95% confidence interval (95% CI). Stepwise shrinkage analysis was applied to identify the best predictive miRNA.

Finally, the ability to predict the status of NCWS was performed using a receiver operating characteristic (ROC) curve (Eng J. ROC analysis: web-based calculator for ROC curves. Baltimore: Johns Hopkins University. Available from: http://www.jrocfit.org.). The true positive rate was plotted versus the false positive rate, using the PC1 values. The area under the curve (AUC) was calculated as a measure of classification model performance.

The described statistical analysis was performed both on biopsies and on PBLs specimens of the validation cohort. For all tests, the threshold for statistical significance was set at p < 0.05. All analyses were performed with the open-source statistical R software (version 3.4.3, The R Foundation for Statistical Computing).

## Results

### Screening and enrollment

We screened in total 144 patients with self-reported gluten/wheat-related symptoms; of those 117 underwent further evaluation according to our study protocol. After screening for CD including a negative esophagogastroduodenoscopy (EGD) and H. pylori status, wheat allergy, and SIBO, in 58 subjects an open GFD was prescribed by an experienced nutritionist for a 6 week-period. At the end of this period, 46 patients were persistently symptom-free, while after dietary gluten re-introduction, using a modified version of the previously administered dietary scheme to introduce wheat protein (equivalent to 10 gr of gluten), 40 showed symptom recurrence and were diagnosed as NCWS. Twenty-four newly diagnosed celiac disease patients and 42 controls showing wheat/gluten independent dyspeptic symptoms were also enrolled.

### Assessment of the expression levels of selected miRNAs in the intestinal mucosa of NCWS patients and controls

To identify miRNAs potentially involved in NCWS, we exploited two commercially available PCR arrays (miScript) designed to the analysis of specific immune-related miRNA. Total RNA extracted from the biopsies of 13 NCWS patients and 17 controls patients with gluten-independent gastrointestinal symptoms (Table 1) were used for quantitative real-time PCR of the miScript arrays. With the aim of limiting the effects of possible changes in reference RNA, the normalization of amplifications relied on six different small RNAs (see methods).

**Fig 1:**
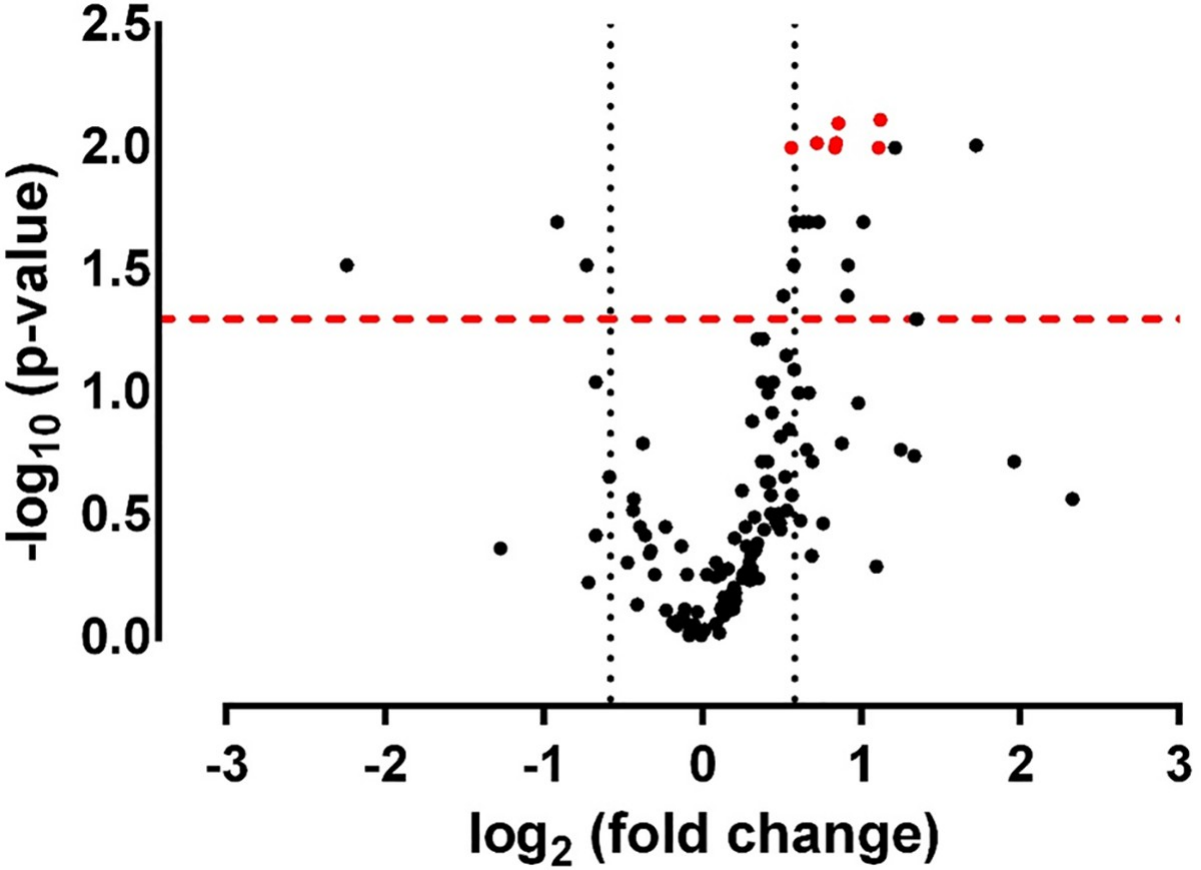
Volcano plot of the Log2 fold changes versus the -Log10 of p values (Control vs NCWS). Total RNA extracted from duodenal biopsies of NCWS patients and control patients affect by gluten-independent dyspeptic symptoms was used to PCR amplify 136 selected miRNA. The fold changes of miRNA expression were calculated according to the 2^-ΔΔCt^ method. Statistical differences between the two groups (control *vs* NCWS) were calculated using a linear regression model, adjusted for age and gender. Vertical dotted lines indicate thresholds of fold changes. The miRNA above the dashed red line have a p<0.05, while those significantly different after FDR correction are highlighted in red.

**Fig 2:**
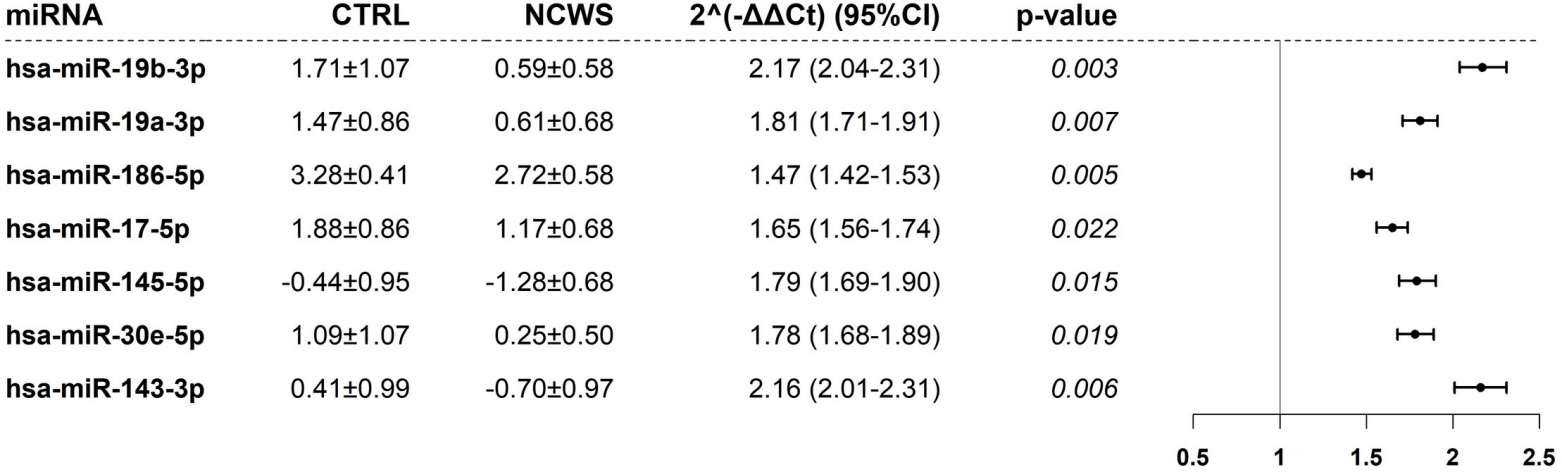
Significant miRNAs after FDR correction in duodenal biopsies of the pilot cohort. The figure reports the ΔCt of miRNA in controls (CTRL) and NCWS patients as well as the fold change (2^-ΔΔCt^) with the relative confidence intervals. The p-value relative to a linear model were adjusted for age and gender. ΔCt are expressed as mean±standard deviation (SD). In the right panel, the fold change values and their confidence intervals are shown graphically.

Differentially expressed miRNAs were selected by volcano plot filtering (fold change ≥ 1.5 and p-value ≤ 0.05) as shown in Fig 1. Note that, in consideration of the general characteristics of the patients’ cohort, statistical significance of the difference between miRNA expression of the two groups was evaluate using a linear model, adjusted for age and gender. After false discovery rate (FDR) correction[40] seven differentially expressed miRNAs were identified in the duodenal biopsies of NCWS patients (Fig 2).

### The expression levels of six miRNA are distinctive of patients affected by NCWS

To confirm the results obtained in the above pilot experiment we decided to assess the expression of the seven differential miRNAs on a larger cohort of patients (validation cohort). To this aim, we used an in-house built quantitative real time PCR instead of the precast miScript miRNA PCR arrays (see methods). The validation cohort consisted of 25 control patients with gluten-independent gastrointestinal problems, and 27 NCWS patients. A complete description of the patients used in this part of the study is reported in Table 1.

Using the statistical approach detailed in the methods, we confirmed the differential expression of 6 miRNAs (Table 2) in this validation cohort. The discriminant analysis provided evidences that these 6 miRNA have a classification accuracy of about 52% in controls and 77.8% in patients with NCWS.

**Table 2:** Significant miRNAs in duodenal biopsies of the validation cohort.

miRNA	CTRL	NCWS	2^-ΔΔCt^ (95%CI)	p-value^b^	CD	2^-ΔΔCt^ (95%CI)	p-value^c^	ANOVA^a^
**hsa-miR-143-3p**	-1.08±0.74	-1.37±0.40	1.22 (1.03–1.45)	*0*.*215*	-0.88±0.81	1.40 (1.17–1.69)	*0*.*019*	*0*.*034*
**hsa-miR-145-5p**	-1.64±1.02	-2.24±0.59	1.51 (1.19–1.93)	*0*.*013*	-1.63±0.66	1.53 (1.28–1.82)	*0*.*013*	*0*.*007*
**hsa-miR-19a-3p**	0.99±1.69	-0.28±0.95	2.40 (1.61–3.58)	*0*.*003*	0.68±1.43	1.95 (1.37–2.75)	*0*.*029*	*0*.*004*
**hsa-miR-19b-3p**	1.14±1.58	-0.08±0.93	2.33 (1.60–3.39)	*0*.*003*	1.05±1.42	2.19 (1.55–3.09)	*0*.*007*	*0*.*002*
**hsa-miR-30e-5p**	0.16±0.84	-0.30±0.62	1.38 (1.12–1.70)	*0*.*046*	0.63±0.69	1.91 (1.59–2.29)	*<0*.*001*	*<0*.*001*
**hsa-miR-186-5p**	2.39±0.81	1.96±0.62	1.35 (1.10–1.65)	*0*.*077*	2.50±0.82	1.45 (1.18–1.79)	*0*.*025*	*0*.*029*

^a^p-value relative to linear model, adjusting for age and gender

^b^ Dunnett’s t post-hoc test CTRL vs NCWS

^c^ Dunnett’s t post-hoc test CD vs NCWS

Data are expressed as mean±standard deviation (SD) of ΔCt

**Fig 3:**
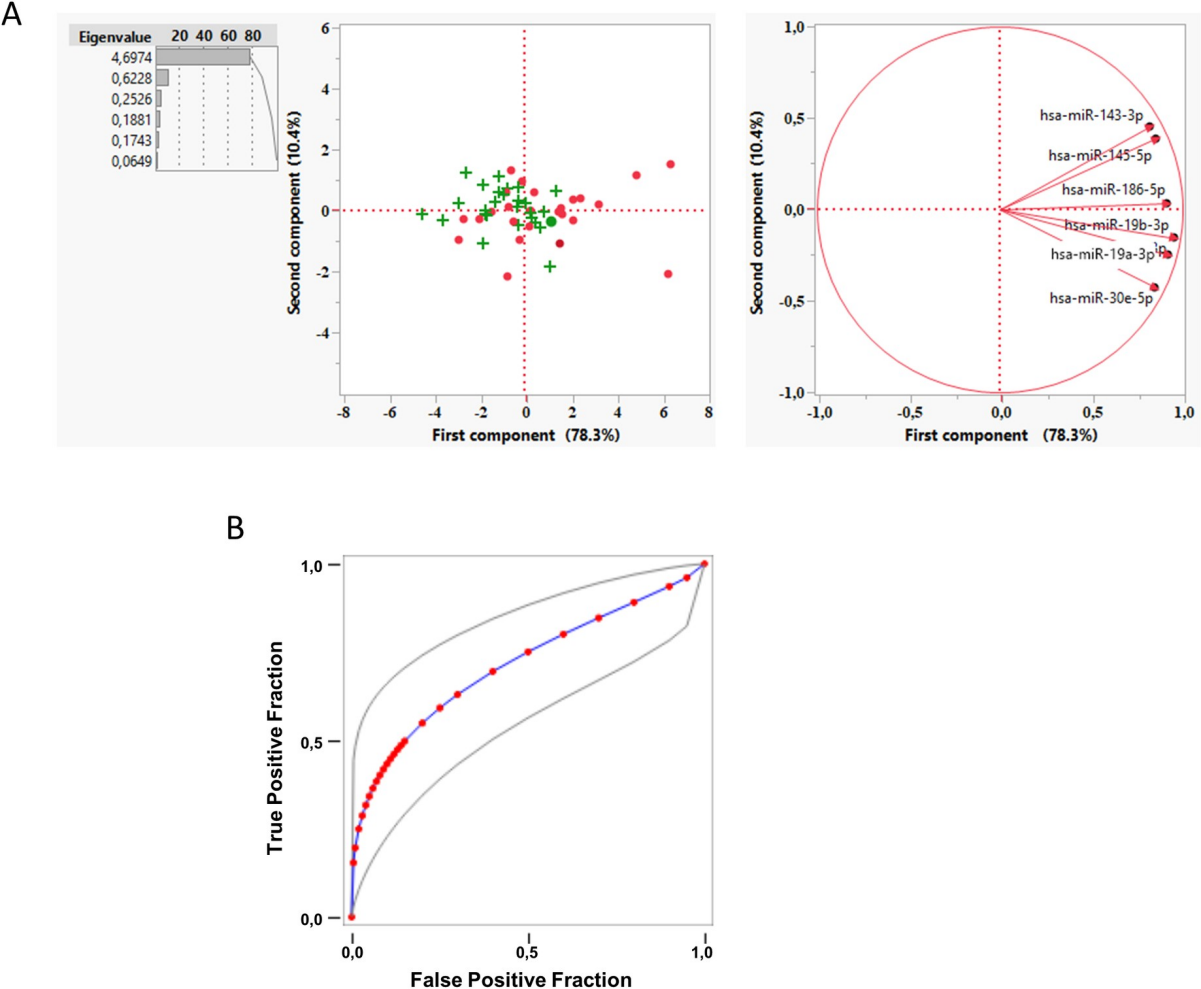
A principal component analysis of miRNA expression levels in duodenal biopsies can classify patients with NCWS and controls. (**A**) A principal component analysis was performed on the ΔCt values for each gene. First component (PC1) was plotted against second component (PC2) to identify genes driving the two components and to explain the percentage variation. • Control group; + NCWS group. (**B**) Fitted ROC curve (blue line) of the first principal component data (red dots). Plotting of the true positive rate versus false positive rate as defined by the PC1 values. AUC = 0.74 ± 0.0702 (p<0.001, Wilcoxon U test). Grey lines: 95% confidence interval of the fitted ROC curve.

In order to reduce the dimensionality of the data we carried out a principal component (PC) analysis of the delta Ct (ΔCt, see methods). The first component explains almost 78.3% of the variance and correlates with NCWS status when compared to control group (Fig 3A). For a one-unit increase in PC1 there was a 35% decrease in the risk of NCWS status (OR = 0.35; 95% CI: 0.16–0.78, p = 0.011). The expression of miRNA have a direct relationship with PC1 values, therefore the odds of NCWS status is greater among patients where these miRNA are more expressed, that is with a lower ΔCt. In contrast, there was no evidence of an association between the PC2 and the NCWS status. The PC1 performance, as a classifier, was estimated from the area under the curve (AUC) of the Receiver Operating Characteristic (ROC), which indicates 74% of probability to classify patients in their group (Fig 3B). Finally, applying a stepwise shrinkage analysis we identified hsa-miR-19b-3p as the most predictive miRNA. The 70.4% of the NCWS patients was reclassified in its own group using a model with only this miRNA.

### The expression of six miRNA can identify/classify patients affected by NCWS and active CD

As described above, NCWS is commonly a diagnosis by exclusion [42]. CD is a well characterised gluten-related disorder and presents a number of signs and symptoms overlapping those of NCWS [43]. Thus, prompted by the above results, we decided to extend our analysis and investigate whether the expression of miRNA might be also useful to distinguish patients with NCWS from those with CD. We therefore examined the expression of the 7 differential miRNAs, identified in the pilot step, in 24 patients affected by CD and with a high degree of intestinal alteration (Table 1). The expression of miRNA was investigated using the in-house real time PCR described above.

Statistical analysis indicated that 6 miRNA are differentially expressed between NCWS and CD (Table 2). The discriminant analysis forestall that the measurement of these 6 miRNA have a classification accuracy of 60% in CD and 81.5% in NCWS patients.

**Fig 4:**
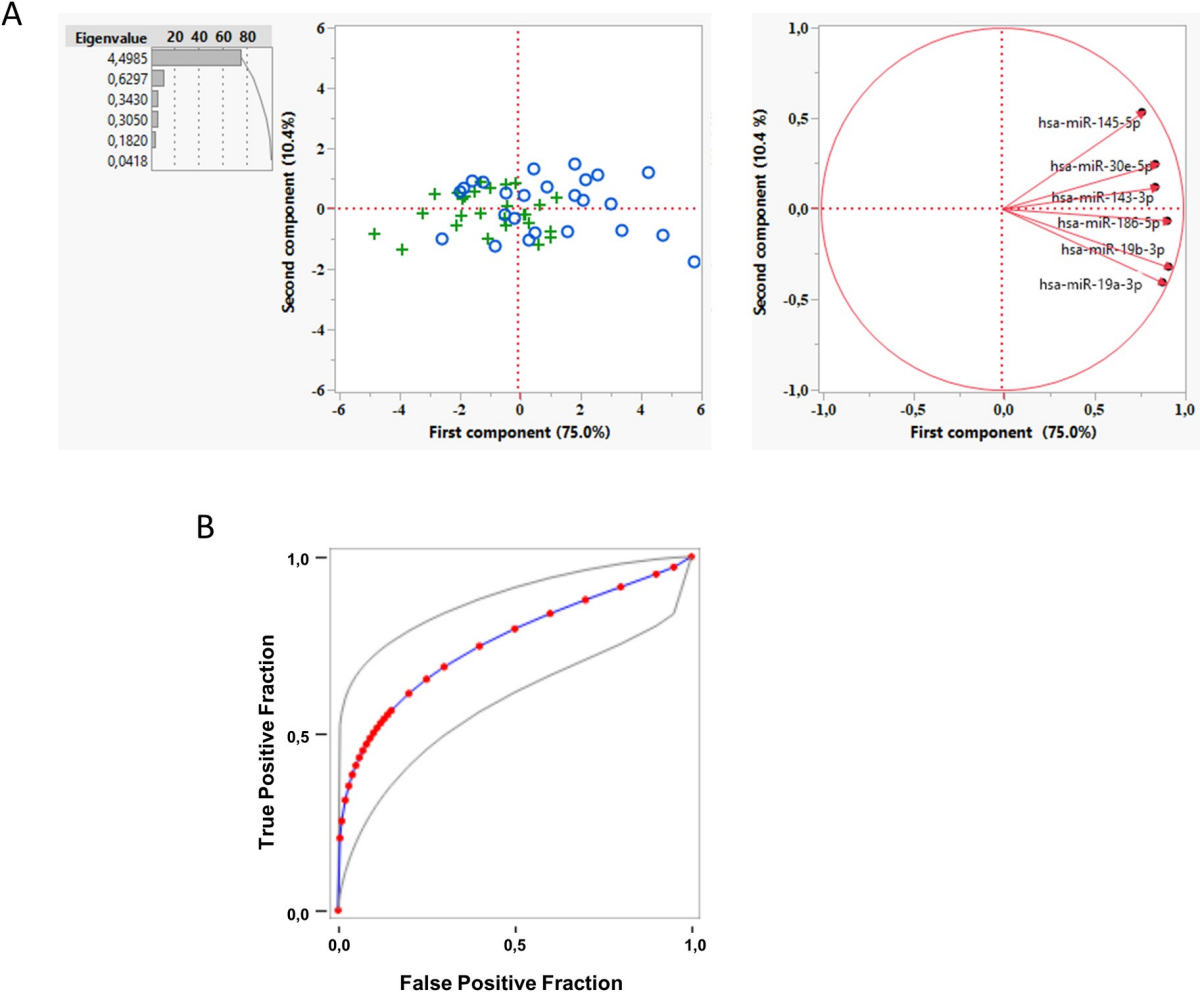
A principal component analysis of miRNA expression levels in duodenal biopsies can classify with NCWS and CD. (**A**) A principal component analysis was performed on the ΔCt values for each gene. First component (PC1) was plotted against second component (PC2) to identify genes driving the two components and to explain the percentage variation. + NCWS group; ◦ CD group. (**B**) Fitted ROC curve (blue line) of the first principal component data (red dots). Plotting of the true positive rate versus false positive rate as defined by the PC1 values. AUC = 0.76 ± 0.0680 (p<0.001, Wilcoxon U test). Grey lines: 95% confidence interval of the fitted ROC curve.

Furthermore, to reduce the number of parameters that recapitulate these data we carried out a PC analysis of ΔCt. PC1 explains 75% of the variance and correlates with NCWS status when compared to CD group (Fig 4A). For a one-unit increase in PC1 there was an estimated decrease of risk of NCWS status (OR = 0.36; 95% CI: 0.16–0.79, p = 0.011). This suggest that the odds of NCWS status is greater among patients where the miRNA are more expressed and thus have a lower ΔCt. Again, there was no evidence of an association between PC2 and NCWS status. The AUC of the ROC curve indicates that PC1 values have 76% of probability of classifying the patients (Fig 4B). According to the stepwise shrinkage analysis, hsa-miR-30e-5p is the most predictive miRNA for the NCWS status. The 74.1% of NCWS patients was reclassified in its own group using a model with only this miRNA.

### The expression levels of six miRNA from peripheral blood leukocytes can be exploited to identify patients affected by NCWS

Based on the above, the measurement of miRNA expression in patients’ intestinal mucosa might be helpful to the diagnosis of NCWS. However, this task involves the implementation of an invasive gastrointestinal endoscopic biopsy. Here, we decided to assess the expression of relevant miRNAs in PBL, as alterations of miRNA expression could be present in other tissues including PBL.

Peripheral blood was collected from 19 NCWS patients and 21 control patients with gluten-independent gastrointestinal symptoms with functional dyspepsia (Table 1). To reduce the effect of external factors on the possible variability of leukocytes population we collected the blood from patients with no obvious signs of infection. However, the number of leukocytes were not statistically different between the patient groups (not shown).

**Fig 5:**
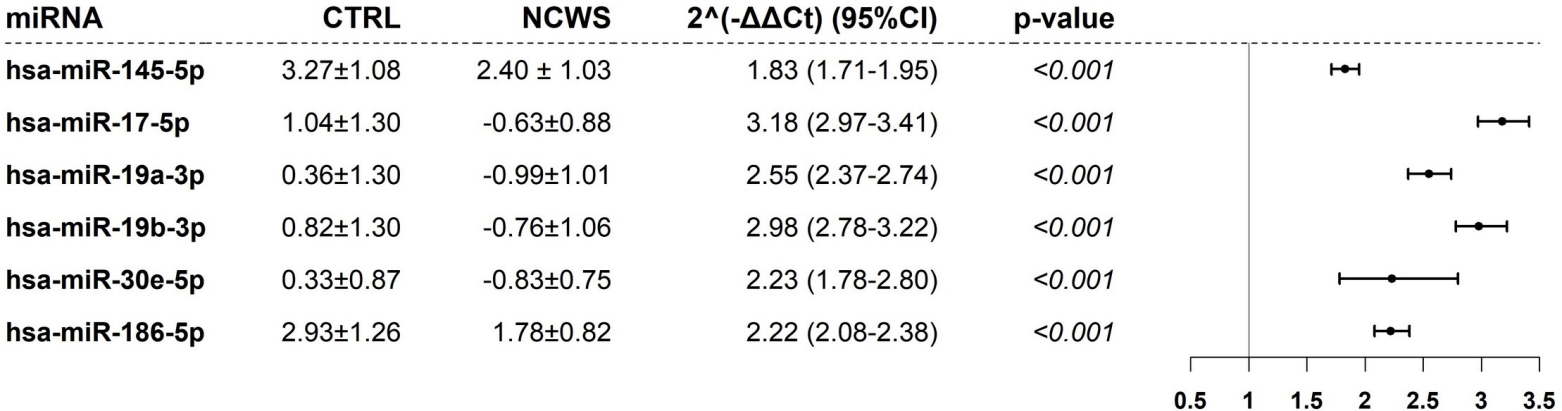
Significant miRNAs in PBL of the validation cohort. The figure reports the ΔCt of miRNA in controls (CTRL) and NCWS patients as well as the fold change (2^-ΔΔCt^) with the relative confidence intervals. The p-value relative to a linear model were adjusted for age and gender. ΔCt are expressed as mean±standard deviation (SD). In the right panel, the fold change values and their confidence intervals are shown graphically.

The expression of the 7 miRNA identified in the pilot analysis were measured in the two groups of patients by in-house real time PCR. Statistical analysis showed that indeed 6 miRNA are differentially expressed between NCWS and controls PBL (Fig 5). The discriminant analysis provided evidences that these 6 miRNA have a classification accuracy of 85.7% in controls and 73.7% in NCWS patients.

**Fig 6:**
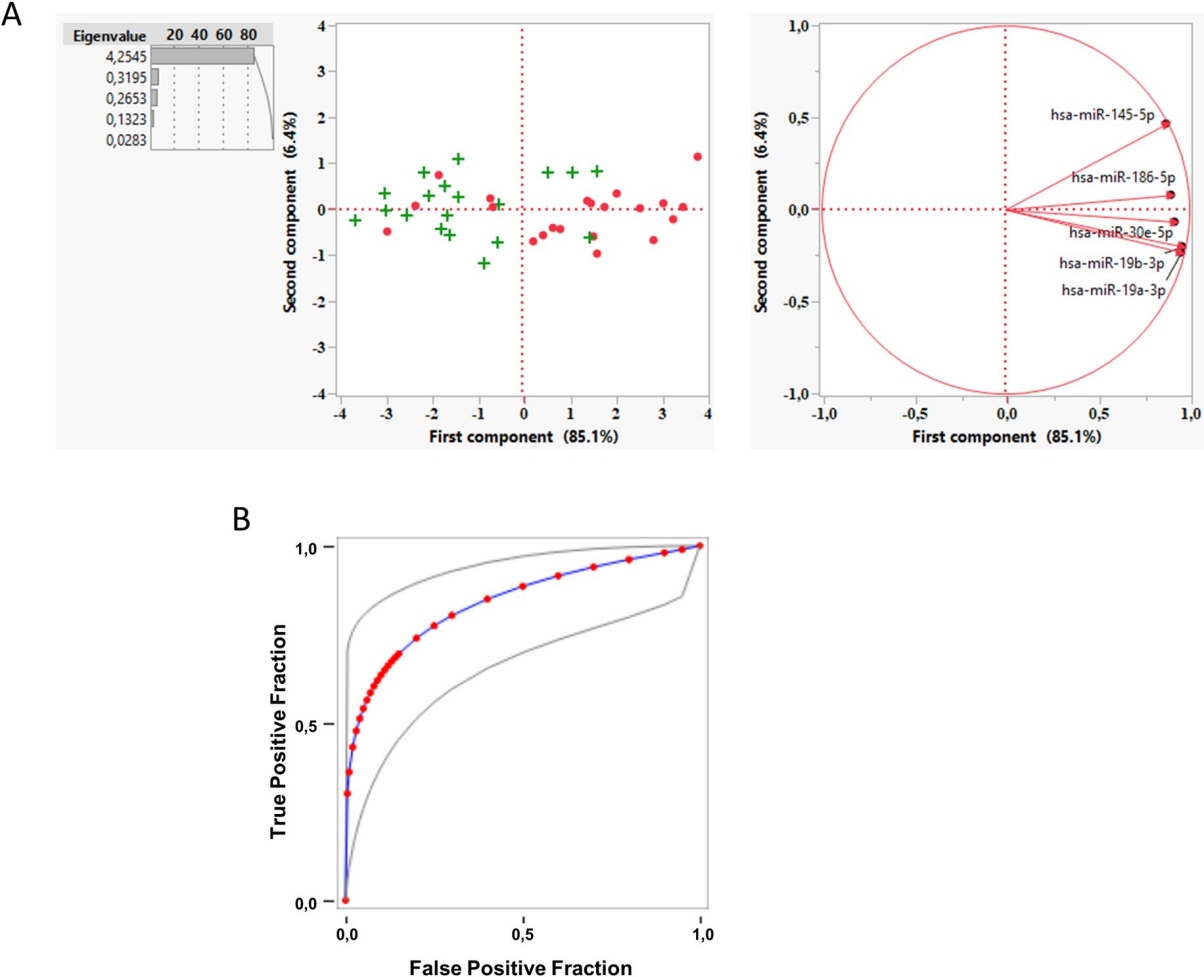
Principal component analysis of miRNA expression levels in PBL can classify patients with NCWS and controls. (**A**) A principal component analysis was performed on the ΔCt values for each gene. First component (PC1) was plotted against second component (PC2) to identify genes driving the two components and to explain the percentage variation. • Control group; + NCWS group. (**B**) Fitted ROC curve (blue line) of the first principal component data (red dots). Plotting of the true positive rate versus false positive rate as defined by the PC1 values. AUC = 0.84 ± 0.0624 (p<0.001, Wilcoxon U test). Grey lines: 95% confidence interval of the fitted ROC curve.

The PC1 explains 85.1% of the variance and correlates with NCWS status when compared to control group (Fig 6A). For a one-unit increase in PC1 there was an estimated 21% decrease in the odds of NCWS status (OR = 0.21; 95% CI: 0.08–0.55, p = 0.001). There was no evidence of an association between PC2 and NCWS status. The AUC of the ROC curve built up with PC1 values indicates that this classifier may recognize about 84% of the patients (Fig 6B). Finally, using the stepwise shrinkage analysis, hsa-miR-30e-5p resulted the most predictive miRNA for NCWS status also in PBL. The 78.9% of NCWS patients was reclassified in its own group using a model with only this miRNA.

## Discussion

In this study we recruited patients affected by NCWS with the aim to define a miRNA signature which can identify these patients and distinguish them from symptomatic controls with functional dyspepsia or CD.

The screening of selected miRNA lead to the identification of seven miRNA (hsa-miR-19b-3p, hsa-miR-19a-3p, hsa-miR-186-5p, hsa-miR-17-5p, hsa-miR-145-5p, hsa-miR-30e-5p, hsa-miR-143-3p) which are significantly overexpressed in the intestinal mucosa of NCWS patients in comparison to control patients affected by gluten-independent gastrointestinal problems and patients with CD. Remarkably, six of these miRNA were found to be overexpressed also in PBL of NCWS patients.

Moreover, it is interesting to notice that hsa-miR-30e-5p was the better predictor of NCWS vs CD in biopsies specimen analysis, while the same miRNA was better predictor of NCWS vs control in PBL. We hypothesise that the expression of a few miRNAs may vary according to the tissue sample used for analysis. Alternatively, the narrow CI shown by hsa-miR-30e-5p in our patients’ cohort may account for this result. However, our analyses were not powered enough to test this hypothesis and future studies with larger population should be planned to specifically answer to this question.

The relevance of our finding is underscored by the current lack of biomarkers and laboratory tests useful to validate the diagnosis of NCWS. Unfortunately, nowadays these diagnoses are based on the evaluation of symptoms changes in response to gluten ingestion, leading people suffering from unspecified gastrointestinal discomfort to start gluten free diet by their own decision. Furthermore, the recent overwhelming upward trend of gluten free diet also acts as a confounding factor. To overcome the lack of specific biomarkers of NCWS and more specifically its gluten-specific variant, it has recently been introduced a specific gluten challenge protocol known as “The Salerno Experts’ Criteria” [17]. This relies on a complex gluten double-blind exclusion/reintroduction protocol, which is potentially applicable, if ever, only in a clinical study setting. Moreover, gluten challenge is positive in only a minority of patients with a clinical phenotype of NCWS[44] suggesting the need of more accurate and objective diagnostic tools, diagnosis based on symptoms and the occurrence of a “nocebo” effect reduces the sensitivity and specificity of these criteria.

At the time of conception of this study, non-celiac wheat/gluten sensitivity diagnosis was based on symptom correlation to gluten consumption not requiring a double-blind confirmation. Although this should be considered a limitation of this study, potentially permitting inclusion of patients with different sensitivities to various wheat components and not only to gluten, it is unclear whether a more stringent double-blind approach would be more effective in recruiting a more homogeneous group of wheat/gluten-sensitive patients. Our approach of including patients reporting only more “classical” gastrointestinal symptoms could lead to easier and more objective identification of NCWS patients. The capability of miRNA patterns to discriminate between groups in this setting, further strengthens this assumption. As differences in clinical presentation of wheat/gluten sensitivity are presently limited to symptom patterns, further studies are needed to determine whether our methodology is equally applicable regardless of prevailing symptoms and whether miRNA patterns could be of further pathophysiological interest in this setting.

This study is, to our knowledge, the first identifying biomarkers of NCWS that, if translated into clinical grounds might contribute to the diagnosis of NCWS.

Micro-RNA are a large family of a few thousands of small non coding RNA capable of regulating gene expression post translationally [45]. Each miRNA can control the expression of hundreds of mRNA and thus they virtually regulate any gene and affect countless cell functions. Experimental evidence linked miRNA with cell proliferation, differentiation, tissue homeostasis and multiple diseases [26]. In human genome, some miRNA family or cluster may share similar regulation and functions [26, 45].

Based on the importance of miRNA in tissue homeostasis and disease development our finding might provide relevant hints about NCWS pathology. Literature data established a link between downregulation of miR-143/145 cluster and development of epithelial cancers including colon cancer [46]. From a physiological standpoint it appears that miR-143/145 are required for the renewal of intestinal epithelium as their knock down heavily impaired intestinal regeneration after injuries [47]. The overexpression of miR-143/145 in the intestine of NCWS patients may indicate an undergoing regeneration process [47]. However, in line with the multiple gene targets of miRNAs, miR-145 was also involved in the negative regulation of inflammation associated with chronic glomerulonephritis, metabolic syndrome and hypoxia induced heart injury [48–50]. Remarkably, miR-145 is upregulated in different autoimmune disease including multiple sclerosis [51–53], systemic lupus erythematosus and primary Sjögren's syndrome [54] suggesting possible autoimmune aspects in the NCWS.

Another important cluster of miRNA is the miR-17/92, which comprises 6 miRNA including the has-miR-17-5p, has-miR-19a-3p and has-miR-19b-3p identified in this study [55]. These miRNA, among the others, are involved in the development of immune cell progenitors (e.g. common myeloid progenitors and macrophage progenitors) and the regulation of innate and adaptive immune functions [56]. Within the innate response, miR-17/92 participate into monocytes differentiation/activation and inhibition of the immunosuppressive role of myeloid-derived suppressor cells [56]. Finally, miR-17/92 have a central role in adaptive immune functions where they promote the differentiation of B cells and various subfamily of T cells including the cytotoxic, Th1, Treg and Th17 [56]. Among the miR-17/92 cluster, miR-17 and miR-19 are key controller of TH17 response [57]. The knockout of these miRNA prevented weight loss in experimental colitis and reduced the clinical score of experimental autoimmune encephalomyelitis (model of multiple sclerosis) [57] indicating an involvement in autoimmune diseases. The overexpression of miR-17 and miR-19 in NCWS suggests a possible dysregulation of Th17 pathway as a pathogenic determinant of NCWS. Along this line, a recent study reported a Th17 signature in NCWS patients [58].

As far as miR-30e is concerned, it may control the activation of natural killer cells, although its involvement in immune response is poorly defined [56]. Notably, miR-30e was found overexpressed in the plasma of patients affected by systemic lupus erythematosus, relapsing remitting multiple sclerosis and paediatric Crohn’s disease, three main autoimmune inflammatory diseases [53, 59, 60]. We hypothesise that this miRNA might be induced by the inflammatory status present in these pathologies or may itself contribute to the development of autoimmune diseases. Lastly, the miR-186, one of the miRNAs upregulated in NCWS, was previously reported as increased in the serum of relapsing remitting multiple sclerosis patients [51, 52].

According to the available information, miRNAs that characterise NCWS are deregulated in several autoimmune diseases possibly because of their role in immune tolerance [51–54, 59, 60]. The miR-17/92 cluster can exemplify these concepts; it impinges on the Th17 response, a subset of T cells that controls acute and chronic inflammation, and contributes to the development of autoimmune disorders [55–57, 61]. Thus, our finding suggests that a dysregulation of Th17 pathway and autoimmune mechanisms may be involved in NCWS pathogenesis, although this calls for future investigations. Ingenuity Pathway Analysis (IPA) confirmed some overlap of our miRNA signatures with idiopathic pulmonary fibrosis, multiple sclerosis, experimental autoimmune encephalomyelitis and class II lupus nephritis. These results suggest that NCWS may share pathogenic pathways with chronic inflammatory disorders.

In conclusion, the present study shows that NCWS patients have distinctive miRNA expression profiles. Differentially expressed miRNA patterns combined with serological and histological exclusion of CD, might represent a future marker for positive diagnosis of this syndrome. However, the introduction of this type of signature in the clinic requires the implementation of additional steps. Firstly, the differentially expressed miRNA must be validated on larger cohorts of patients selected from different laboratories according to the most advanced diagnostic criteria. Secondly, it would be useful to understand if miRNAs are basically altered, in patients with NCWS, regardless of the diet or are altered as a consequence of the administration of a gluten-containing diet.

## Abbreviations

ATI: amylase trypsin inhibitor
CD: celiac disease
EGD: esophagogastroduodenoscopy
EmA: anti-endomysial antibodies
FC: fold change
FDR: false discovery rate
FODMAPs: fermentable oligo-, di-, monosaccharide
GFD: gluten free diet
HLA: human leukocyte antigen
IPA: Ingenuity Pathway Analysis
hsa-: homo sapiens
LPS: lipopolysaccharide
miRNA: microRNA
NCWS: non-celiac wheat sensitivity
NHANES: National Health and Nutrition Examination Survey
OR: odds ratio
PBL: peripheral blood leukocytes
PC: principal component
AUC: area under the curve
RISC: RNA-induced silencing complex
ROC: receiver operating characteristic
SIBO: small intestinal bacterial overgrowth
TLR: toll like receptor
IL: interleukin
tTG: tissue transglutaminase
