# Correction: An explorative study identifies miRNA signatures for the diagnosis of non-celiac wheat sensitivity

**DOI:** 10.1371/journal.pone.0231273

**Published:** 2020-03-27

**Authors:** The PLOS ONE Staff

There are errors in the Funding Statement. The publisher apologizes for these errors. The correct Funding statement is: This work was supported by Fondazione Celiachia Onlus, Italy, Grant n° 046_FC_2013.
